# Optimal single sampling time-point for monitoring of praziquantel exposure in children

**DOI:** 10.1038/s41598-021-97409-x

**Published:** 2021-09-09

**Authors:** Rajabu Hussein Mnkugwe, Eliford Ngaimisi Kitabi, Safari Kinung’hi, Appolinary A. R. Kamuhabwa, Omary Mashiku Minzi, Eleni Aklillu

**Affiliations:** 1 Department of Clinical Pharmacology, School of Medicine, Muhimbili University of Health and Allied Sciences, Dar es Salaam, Tanzania; 2 Division of Clinical Pharmacology, Department of Laboratory Medicine, Karolinska University Hospital Huddinge, Karolinska Institutet, Stockholm, Sweden; 3 Office of Clinical Pharmacology, Division of Pharmacometrics, Food and Drugs Administration, Silver Spring, MD USA; 4 National Institute for Medical Research, Mwanza Research Centre, Mwanza, Tanzania; 5 Department of Clinical Pharmacy and Pharmacology, School of Pharmacy, Muhimbili University of Health and Allied Sciences, Dar es Salaam, Tanzania

**Keywords:** Paediatrics, Public health, Therapeutics, Paediatric research, Medical research

## Abstract

Praziquantel pharmacokinetics studies in schistosomiasis infected children are scarce partly due to the challenges/complexity of intensive blood sampling in the target population. This study was aimed to investigate the optimal single sampling time-point for monitoring praziquantel exposure. This was intensive pharmacokinetic study conducted among 32 *Schistosoma mansoni* infected children treated with an oral standard single-dose 40 mg/kg praziquantel. Plasma samples were collected at 0, 1, 2, 4, 6 and 8 h post-praziquantel administration. Quantification of praziquantel and its enantiomers (*R-* and *S-*praziquantel) concentrations was done by Liquid chromatography-tandem mass spectrometer (LC–MS/MS). The correlation between area under the plasma concentration–time curve from 0 to 8 h (AUC_8_) and plasma concentrations at each specific sampling time-point was determined by Pearson’s correlation coefficient (r^2^). The median age (range) of the study population was 12.5 years (10–17). The study participants were 17 males and 15 females. Both total praziquantel and its enantiomers (*R-* and *S-*praziquantel) displayed a wide inter-individual pharmacokinetic variability. Regression analysis indicated that, plasma concentrations collected at 4 h post-dose had a significantly highest correlation with the AUC_8_ for both total praziquantel (r^2^ = 0.81, p < 0.001) and *S-*praziquantel (r^2^ = 0.84, p < 0.001) than any other sampling time-point; while for *R-*praziquantel, plasma concentrations collected at 6 h sampling time-point had a significantly highest correlation with the AUC_8_ (r^2^ = 0.79, p < 0.001) than any other sampling time-point. Four hours sampling time-point post-praziquantel administration is ideal optimal single sampling time-point for therapeutic monitoring of total praziquantel exposure while 6 h sampling time-point is suitable for monitoring of a pharmacologically active *R-*praziquantel enantiomer.

## Introduction

Schistosomiasis is still a public health challenge in Sub-Saharan Africa (SSA). The disease causes significant morbidity and mortality especially among children in endemic countries^1^. Over 250 million people are infected with schistosomiasis worldwide and children are the most affected^2^. Preventive chemotherapy by mass praziquantel administration targeting school children is the core control strategy implemented in endemic countries and > 90% of those requiring treatment for schistosomiasis live in SSA^3^. Despite the repeated rounds of mass praziquantel administration, schistosomiasis remain a public health problem in SSA including Tanzania^4,5^.

Praziquantel is a pyrazino-isoquinoline derivative, which has a rapid and complete absorption (> 80%) following its administration. Its systemic bioavailability is usually low and varies between individuals^6^. Praziquantel is distributed throughout the body and is highly bound to plasma protein (about 80%), mostly to albumin^6^. It undergoes an extensive enantioselective first pass metabolism in the liver by cytochrome P450 (CYP450) enzymes including CY3A4/5, CYP2C19, CYP2C9 and CYP1A2 to form several hydroxylated metabolites^6,7^. The major metabolite in humans is *trans*-4-hydroxylated praziquantel, which possess antischistosomal activity^8^. The fact that praziquantel metabolism is entirely via CYP450, its pharmacokinetics is susceptible to variability due to inter-individual pharmacogenetics differences and also risk of drug–drug interactions^6^.

Praziquantel treatment has been associated with varying cure rates, incidence, and profile of adverse events^9–11^. This is partly due to the fact that praziquantel displays a wide inter-individual variability in its pharmacokinetic parameters in the population^12^. The reported adverse events following praziquantel treatment have been linked to an increased risk of treatment non-compliance in the target population and hence low treatment coverage^13^. High incidence of adverse events and variability in praziquantel plasma concentration necessitate the need for assessing praziquantel exposure following treatment^11^. Additionally, praziquantel displays limited efficacy against juvenile worms^14^ and a threat of drug resistance has been reported in previous studies^9^. Therefore, the need for an alternative treatment regimen for the treatment and control of schistosomiasis is imminent. Recently, a combination of praziquantel and dihydroartemisinin-piperaquine produced superior efficacy than praziquantel alone for the treatment of schistosomiasis^15^. Furthermore, the combination of praziquantel and dihydroartemisinin-piperaquine produced an increased systemic praziquantel exposure without affecting the overall safety in the target population^16^. On the other hand, low praziquantel plasma levels have been reported in healthy volunteers who received co-administration of anti-tuberculous drugs and praziquantel^17^, and therefore, this drug–drug interaction may result into poor treatment efficacy in patients. Drug–drug interactions can affect systemic praziquantel levels and hence cure rate, incidence, and profile of adverse events^11^. Drug–drug interactions resulting from both the use of combination chemotherapy and concomitant drugs administration further warrants the need for monitoring of praziquantel exposure, particularly in children^18^.

To study the performance and safety of praziquantel in individual patients and during mass drug administration (MDA), its systemic exposure or bioavailability needs to be examined. Area under the concentration–time curve (AUC) is a pharmacokinetic parameter used to reflect the systemic bioavailability or exposure of the drug. Currently, intensive blood sampling at different time-points post-praziquantel administration is common practice used in pharmacokinetic studies and in clinical trials to determine the AUC and maximum plasma concentration achieved (C_max_) as markers of systemic drug exposure^12,17,19,20^. However, intensive blood sampling is challenging not only to researchers but also to study participants who are mostly children^21,22^. Furthermore, the amount of blood needed to be withdrawn during entire sampling period partly accounts for the most stress and anxiety experienced by the children.

In other infectious diseases such as malaria, single time-point plasma sample collected at day 7 has been well established to predict the long-acting antimalarial drug exposure and cure at 28 days follow-up^23–25^. Similarly, single blood sample collected at 2 h post-drug administration, better reflects the systemic exposure of an immunosuppressive cyclosporine and better predicts clinical outcomes of the patients in organ transplant^26^.

The antischistosomal activity of praziquantel is correlated to its systemic exposure (i.e., AUC) rather than C_max_ achieved^6,12^. To calculate AUC, multiple blood sampling following drug administration is required to estimate the full concentration–time curve. However intensive sampling at a given time interval from all children at the same time present ethical and practical barriers. Therefore, plasma concentration collected at a single sampling time-point which better correlates with the AUC can serve as a surrogate marker for praziquantel systemic bioavailability and can predict schistosomiasis treatment outcomes. The optimal single sampling time-point will also mitigate the challenges of intensive blood sampling and hence increases praziquantel exposure monitoring following treatment, particularly among children^27^. To our knowledge, no study has investigated an optimal single sampling time-point as a surrogate marker to accurately predict systemic praziquantel exposure in children infected with trematodes or cestodes infections for which praziquantel is a treatment of choice. The present study examined the optimal single sampling time-point as a surrogate marker of praziquantel bioavailability monitoring in children.

## Results

### Patient’s and baseline characteristics

A total of 32 *Schistosoma mansoni* infected children were treated with an oral standard single dose praziquantel (40 mg/kg) and completed this study. The study participants were 17 males and 15 females. The median age (range) of the study population was 12.5 years (10–17). The median weight and height of the study participants were 32.5 kg and 140.9 cm, respectively. Most of the children had moderate to heavy *Schistosoma mansoni* infection (Table 1).

**Table 1:** Patient’s and baseline characteristics of the study participants.

Variables	Frequency/value
**Age (years)**	
Median (IQR)	12.5 (12–14)
**Sex**	
Male N (%)	17 (53.1%)
Female N (%)	15 (46.9%)
**Weight (kg)**	
Median (IQR)	32.5 (30.7–34.9)
**Height (cm)**	
Median (IQR)	140.9 (138.8–144.3)
**Infection intensity**	
Light infection N (%)	9 (28.1%)
Moderate infection N (%)	11 (34.4%)
Heavy infection N (%)	12 (37.5%)

*IQR* Interquartile range.

### Pharmacokinetics of praziquantel and its enantiomers in the study population

**Figure 1:**
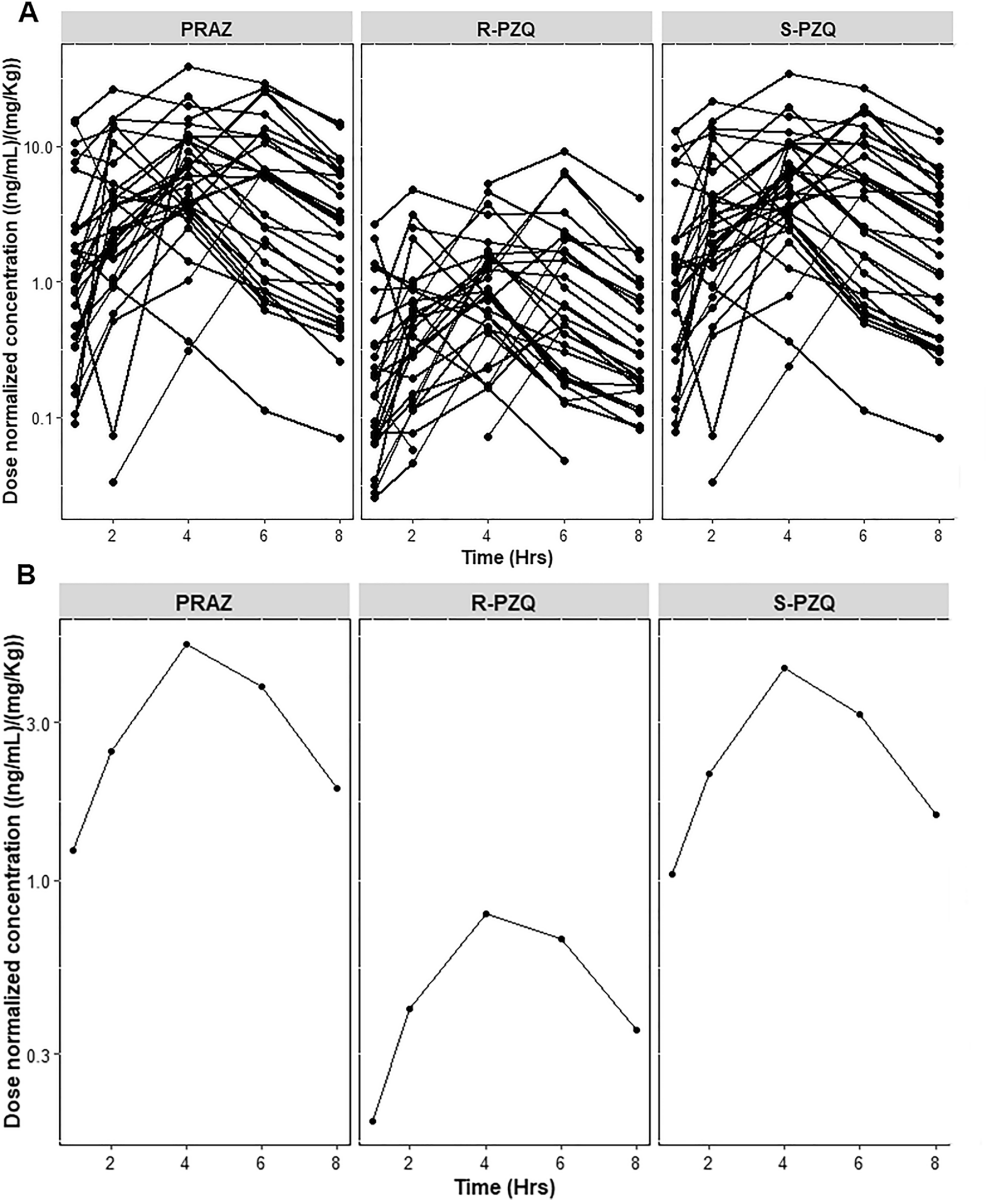
Dose normalized concentration–time profile of total praziquantel (PRAZ), R-praziquantel (R-PRAZ) and S-praziquantel (S-PRAZ) for each study participant (**A**), and the overall mean concentration–time profile plot (**B**).

Changes in total praziquantel and its enantiomers plasma concentrations was monitored at 0, 1, 2, 4, 6 and 8 h after drug administration. The pharmacokinetics parameters of total praziquantel, *R-*praziquantel and *S-*praziquantel are presented in Table 2. The C_max_ in ng/mL for both total praziquantel and enantiomers were achieved at 3.5 h post-dose. The AUC_8_ for total praziquantel was 1254.4 ng h/mL. The AUC_8_ for *S-*praziquantel was higher than that for *R-*praziquantel (Table 2**)**. There was a wide inter-individual variability in pharmacokinetic parameters of both total praziquantel and its enantiomers as presented in Table 2 and Fig. 1.

**Table 2:** Pharmacokinetics parameters of total praziquantel, *R-*praziquantel and *S-*praziquantel expressed as dose-normalized geometric mean and coefficient of variation (CV %).

	Pharmacokinetic parameters				
	AUC_∞_ (ng h/mL)	AUC_8_ (ng h /mL)	C_max_ (ng/mL)	half-life (t_1/2_) (h)	T_max_ (h)
Total PZQ	1281.9 (106.9)	1254.4 (111.6)	346.4 (95.2)	2.2 (47.5)	3.5 (61.8)
*R-*praziquantel	186.9 (84.8)	187.3 (143.7)	56.6 (119.6)	2.0 (35.9)	3.5 (62.8)
*S-*praziquantel	1035.2 (120.0)	1036.9 (114.6)	282.6 (96.3)	2.1 (43.5)	3.5 (61.8)

*AUC* area under concentration–time curve, *C*_*max*_ maximum plasma concentration, *T*_*max*_ time to reach C_max_.

### Correlation between plasma concentrations at different sampling time-points and AUC_8_

**Figure 2:**
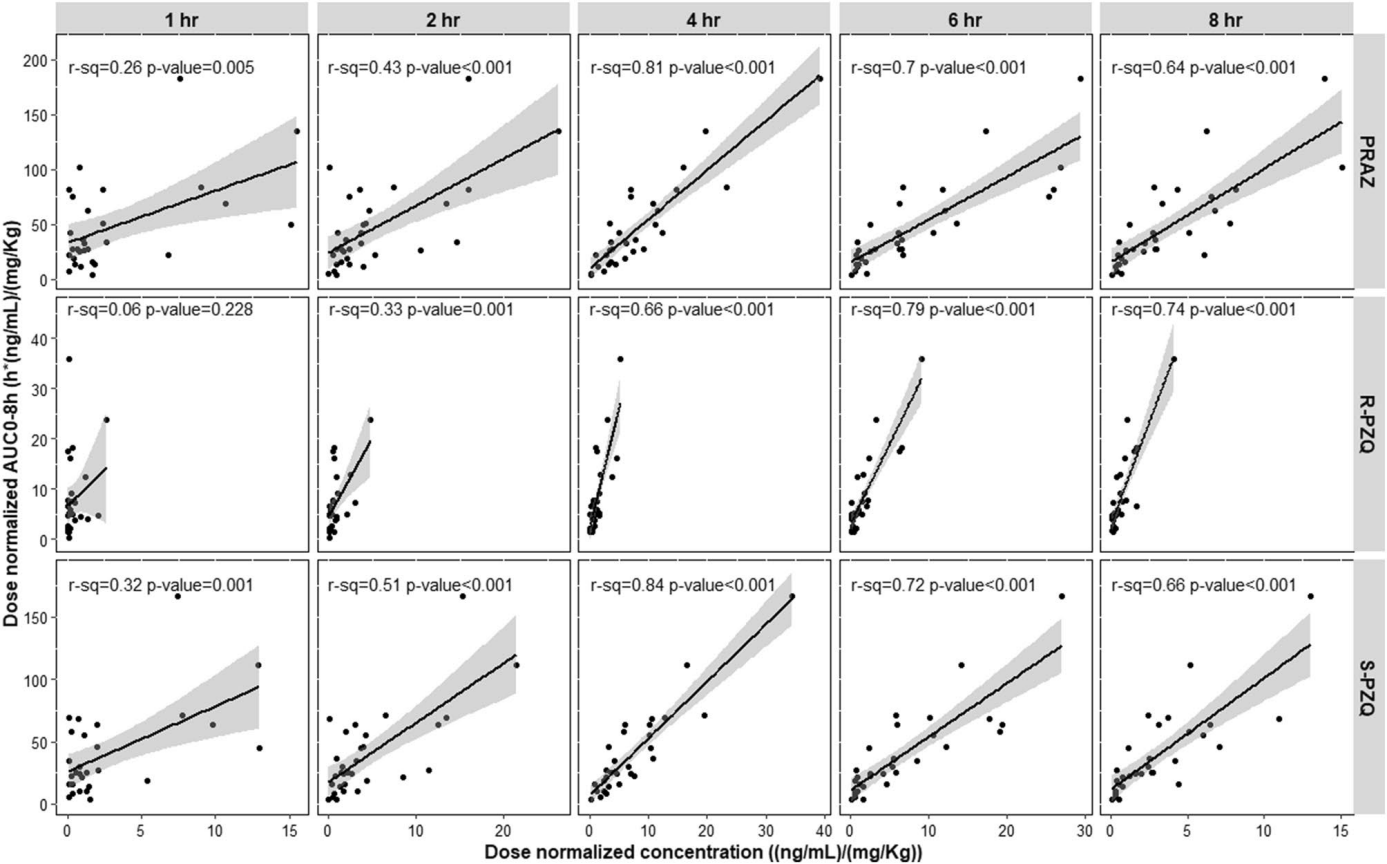
Scatter plot of the correlation between plasma concentrations at each specific sampling time-point and AUC_8_ for total praziquantel (PRAZ), R-praziquantel (R-PZQ) and S-praziquantel (S-PZQ) (*hr* hour).

**Figure 3:**
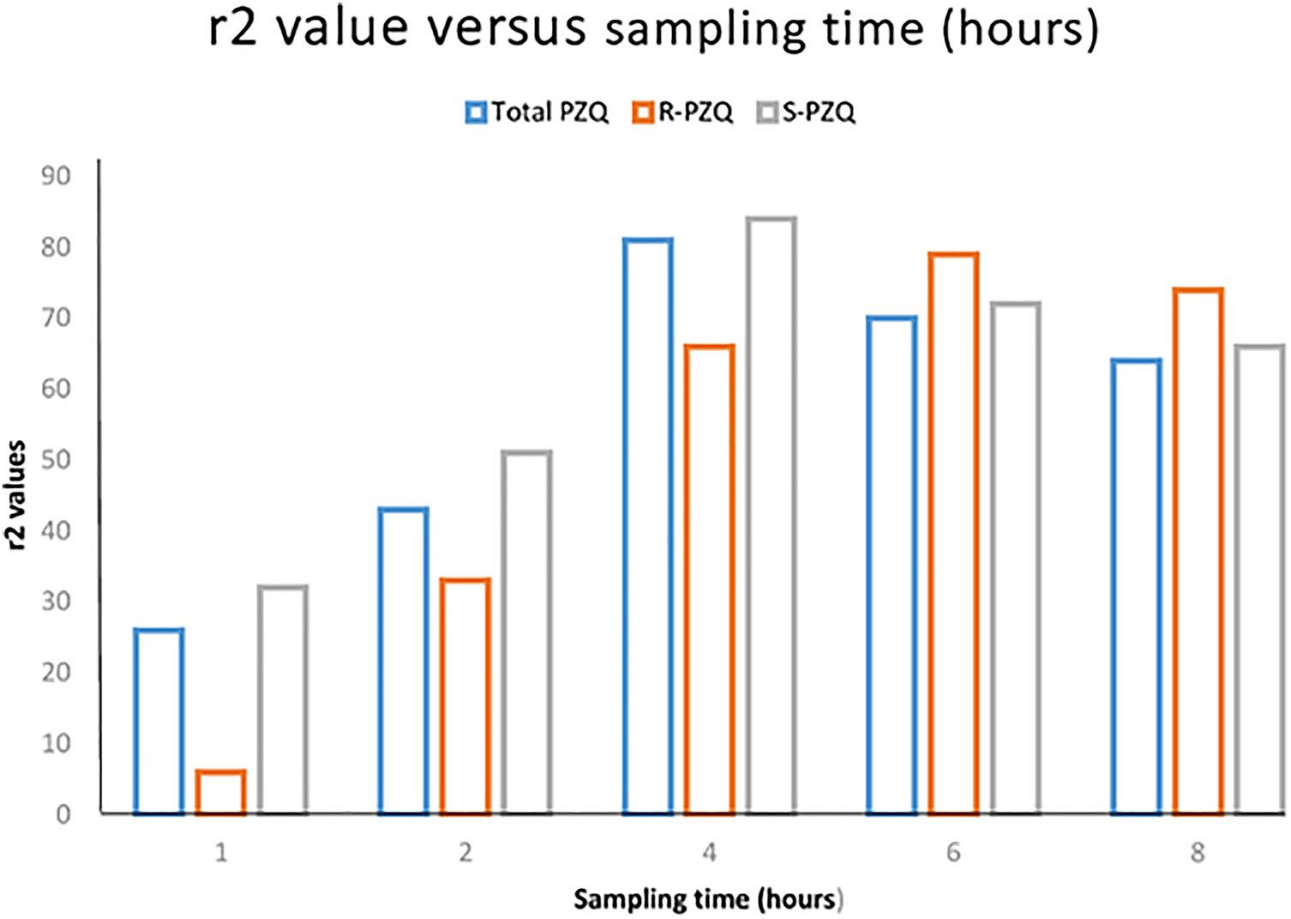
Correlation between plasma concentrations at different sampling time-points and AUC_8_ for total praziquantel (total PZQ), R-praziquantel (R-PZQ) and S-praziquantel (S-PZQ) as determined by Pearson correlation coefficient (r^2^).

The r^2^ values were calculated for the correlation between plasma concentrations collected at each specific time-point (ng/mL) and AUC_8_ (ng h/mL) (Fig. 2). According to the regression analysis, the r^2^ values varied significantly across the plasma concentrations collected at different sampling time-points for both total praziquantel and its enantiomers (Figs. 2, 3). The r^2^ values of the plasma concentrations collected at 4 h sampling time-point for total praziquantel (r^2^ = 0.81, p < 0.001) and *S-*praziquantel (r^2^ = 0.84, p < 0.001) were significantly higher than the rest of the sampling time-points. For *R-*praziquantel, the r^2^ value of the plasma concentration collected at 6 h sampling time-point were significantly higher than the rest of the sampling time-points (r^2^ = 0.79, p < 0.001) (Figs. 2, 3).

## Discussion

We investigated the optimal single sampling time-point for monitoring of praziquantel exposure among schistosomiasis infected children who were treated with single dose praziquantel. Intensive blood sampling over several hours is a common practice in pharmacokinetics studies and clinical trials for the assessment of praziquantel exposure^12,19,20^. Lack of single optimal blood sampling time-point for monitoring of the praziquantel exposure partly contributes to the scarcity of pharmacokinetic data among treated children. The antischistosomal activity of praziquantel depends on the drug exposure i.e. AUC^6^; therefore, sampling time point whose plasma concentrations better correlate with AUC would serve as a pharmacokinetic marker for monitoring praziquantel bioavailability. In malaria treatment using artemisinin-based combination therapy (ACTs), plasma concentrations collected at day 7 for piperaquine or lumefantrine is a marker of antimalarial drug exposure and a predictor of malaria cure^25^. In this study, intensive blood samples were collected at 0, 1, 2, 4, 6 and 8-h post-drug administration, and the AUC_8_ was calculated.

The key findings from the present study include (1) both total praziquantel and its enantiomers display a wide inter-individual variability in pharmacokinetic parameters and (2) plasma concentrations collected at 4 h post-praziquantel administration had a significantly highest correlation (r^2^ value) with AUC_8_ than any other time-points for total praziquantel while plasma concentrations collected at 6 h post-praziquantel had a significantly highest correlation (r^2^ value) with AUC_8_ for a pharmacologically active *R-*praziquantel. To the best of our knowledge, this is the first study to examine the optimal single sampling time-point for monitoring of praziquantel and its enantiomers exposure in children.

In this study, both total praziquantel and its enantiomers exhibited a wide pharmacokinetic variability (Fig. 1) similar to reports from previous studies^12,19^. Based on the literature, most of the praziquantel pharmacokinetics variability is unexplained^12^. However, factors such as differences in age and weights of the study participants (mostly children), food effect, intestinal diseases, and a wide spectrum of CYP450 metabolizing enzymes involved in biotransformation of praziquantel (extensive first pass effect), including CYP3A, CYP1A2, CYP2C9 and CYP2C19 have been speculated^12,19^. CYP enzymes relevant for praziquantel metabolism display wide between-individual and population variation in enzyme activities partly due to pharmacogenetics variations^28–30^ and also display an enantioselective catalytic activity^7^. The difference in CYP enzymes catalytic activity towards praziquantel enantiomers may also account for the observed pharmacokinetics variability. Therefore, there is a need for pharmacogenetics studies of praziquantel and its relevance on plasma concentrations among treated children^11^.

Our study show that plasma concentrations collected at 4 h post-dose is the best single sampling time-point for therapeutic drug monitoring of total praziquantel exposure among treated children. The Pearson’s correlation coefficient (r^2^ value) between plasma concentrations collected at 4 h post-dose and AUC_8_ was significantly higher than any other sampling time-point (r^2^ = 0.81) (Figs. 2, 3). The 4-h post dose sampling point also corresponds well to the T-max for total, R- and S-praziquantel. Therefore, based on these findings, the 4 h sampling time-point following praziquantel administration can be used as a marker of systemic drug exposure or bioavailability among treated children (Fig. 1). The use of this single sampling time-point will mitigate the challenges of intensive blood sampling for assessment of praziquantel exposure especially in children^21,22^.

On the other hand, for *R-*praziquantel, plasma concentrations collected at 6 h post-dose had significantly higher correlation with AUC_8_ than any other sampling time-point (r^2^ = 0.79) (Figs. 2, 3). Therefore, for studies targeting *R-*praziquantel, plasma samples collected at 6 h post-dose gives the best correlation with AUC and would be a suitable single sampling time-point for monitoring of its exposure following praziquantel administration. *R-*praziquantel is the pharmacologically active enantiomer and contributes to the antischistosomal activity of praziquantel^11,19^. Recently, there has been a move to produce a monoenantiomeric *R-*praziquantel formulation for the treatment and control of schistosomiasis in children^11^. *R-*praziquantel formulation will reduce the tablet size and also the bitter taste of the drug that were contributed by the *S-*praziquantel^31^ and hence better treatment compliance among children. Once the *R-*praziquantel formulation is approved for use, a 6-h post-dose sampling time-point is ideal for assessing its systemic exposure following administration.

Future praziquantel pharmacokinetic studies should focus on calculating the cut-off point for the plasma concentrations at 4 h sampling time-point for total praziquantel and 6 h sampling time-point for *R-*praziquantel in order to predict the clinical outcome of schistosomiasis treatment (i.e. cure rate) at 14–21 days follow-up according to the WHO guidelines^32^ and other parasitic infections of which praziquantel is a treatment of choice. In malaria treatment using ACTs, a cut off ≥ 200 ng/mL of day 7 lumefantrine concentration is associated with malaria cure at 28 days follow-up^25^.

PZQ is co-administered with albendazole for the control and treatment of intestinal schistosomiases and soil transmitted helminths (STH) respectively in MDA campaign to all school-age children without prior screening in endemic areas. In our study, participants were screened for intestinal schistosomiases as having *Schistosoma mansoni* infection was the inclusion criteria. Participants were not screened for STH, and this may be considered as study limitation. Although our study finding reflect the practical situation in sub-Saharan Africa where STH and schistosomiasis are co-endemic, STH co-infection may possibly influence PZQ metabolism. As the study participants had heavy-to-moderate *Schistosoma mansoni* infection intensity, any impact of STH co-infection on hepatic PZQ metabolism may be insignificant but cannot be ruled out. Therefore, the impact of STH coinfection on PZQ pharmacokinetics in co-infected children remains to be explored in controlled large sample size studies.

In conclusion, since monoenantiomeric *R-*praziquantel formulation is not yet approved for clinical use, plasma concentrations collected at 4 h post-dose is the ideal optimal single sampling time-point for monitoring of praziquantel bioavailability in children infected with cestodes and trematodes including schistosomiasis, where praziquantel is a treatment of choice.

## Materials and methods

### Study design and population

This was a single-arm pharmacokinetic study aimed at examining the optimal single sampling time-point suitable for monitoring of praziquantel bioavailability during treatment of schistosomiasis in children. A total of 32 *Schistosoma mansoni* infected children (aged 10–17 years) were recruited in this study between May 2017 and January 2018. The enrolled participants were randomly selected from a parallel study assessing the efficacy and safety of single dose praziquantel (40 mg/kg body weight) for the treatment of intestinal schistosomiasis^33^. Hemoglobin concentration (Hb conc) was determined from finger prick blood sample using a HemoCue Hb 201+ machine (HemoCue AB Angelholm, Sweden) to exclude participants with severe anemia (defined as Hb conc < 8 g/dL). As intensive pharmacokinetic study requires multiple blood sampling, laboratory tests that required additional blood sampling such us liver and kidney function test and complete blood count were not done for ethical reasons (to reduce the number and volume of blood samples collected from children, a vulnerable population). However, clinical and physical examinations (i.e. jaundice, edema, pallor etc.) were done before treatment to ensure that the participants had no liver or kidney problems or any other chronic diseases.

Ethical approval for this study was obtained from the National Institute for Medical Research, Dar es Salaam, Tanzania (Ref. No. NIMR/HQ/R.8a/Vol.IX/2343), Muhimbili University of Health and Allied Sciences Institutional Review Board (Ref. No.2016-5-25/AEC/Vol.X/03) and Stockholm Ethics Committee (Ref. No. 2020-00845). This study was conducted in accordance with the Declaration of Helsinki. Written informed consent was obtained from parents/guardians and assent from eligible children.

### Stool samples examination

Two fresh stool samples were collected and analyzed by Kato-Katz technique for screening of *Schistosoma mansoni* infection both at pre- and post-treatment as reported previously^4,33^. Infection intensity was categorized as light infection (epg 1–99), moderate infection (epg 100–399) and heavy infections (epg ≥ 400)^4^.

### Therapeutic procedures and blood sampling

Following a pre-treatment meal, study participants were treated with an oral single dose 40 mg/kg body weight of praziquantel (Praziquantel 600 mg/tablet, Batch BZ6043, S Kant Health Care Ltd, India) as a direct observed treatment (DOT)^33^. Food has been shown to increase the bioavailability of praziquantel^6^. After drug administration, serial blood samples (2 mL) were collected at 0, 1, 2, 4, 6 and 8 h from each participant using an indwelling sterile catheter inserted and maintained in the forearm vein. The withdrawn blood samples in heparinized tubes were immediately centrifuged at 1000 rpm for 10 min to obtain plasma, and aliquots were kept at − 80 °C freezer until analyzed.

### Chemicals and reagents

Racemic (rac) praziquantel was obtained from Merck (Darmstadt, Germany). An 11-fold rac-deuterated-praziquantel (rac-praziquantel-d11) as an internal standard, *R-*praziquantel and *S-*praziquantel were purchased from Toronto Research Chemicals (Toronto, Ontario, Canada). Chemicals such as acetone, acetonitrile, ammonium acetate, isopropanol, methanol, and acetic acid of mass spectrometry (MS) grade were acquired from Merck (Darmstadt, Germany). Ultrapure MilliQ water was prepared using a Milli-Q water purification system (Merck Millipore, MA, USA). Blank human plasma was supplied by the local blood bank of the Karolinska University Hospital Huddinge (Stockholm, Sweden).

### Quantification of R- and S-praziquantel

Quantification of plasma concentrations of R- praziquantel, and S-praziquantel was done using LC/MS/MS as described previously^16^. Briefly, plasma calibration samples were freshly prepared by spiking blank plasma samples with rac-praziquantel and were included in each analytical run. Quality control (QC) samples were also prepared by spiking plasma blanks with known concentrations to obtain low (QCL), medium (QCM), and high (QCH) samples for both *R-*praziquantel and *S-*praziquantel. The quantification range of the method was 1–1500 ng/mL for both *R-*praziquantel and *S-*praziquantel.

For extraction of analytes of interest, 100 µL of plasma samples went through protein precipitation with 200 µL of internal standard solution (50 ng/mL of rac-praziquantel-d11 in methanol) and then vortexed for 10 s followed by centrifugation for 5 min at 2100×*g*. Thereafter, 150 µL of the supernatant was diluted with 75 µL MilliQ water and 5 µL was injected into the LC–MS/MS system. The chromatographic system was using a Chiralpak AGP 2.0 × 100 mm, 5 µm, column (Chiral Technologies Europe, Illkirch, France) with 10 mM ammonium acetate: isopropanol 98:2 (v/v) pH 8 as mobile phase with a flow rate of 0.3 mL/min. The chromatographic run was 22 min, and with use of the parallel two channel capacity, injection to injection time was 11 min. *R-*Praziquantel eluted first followed by *S-*praziquantel with a difference of 1.9 min. Total praziquantel concentrations were obtained by adding *R-*praziquantel and *S-*praziquantel final concentrations. The analytical method was validated according to the European Medicines Agency Guideline on bioanalytical method validation^34^.

### Pharmacokinetic analysis

Noncompartmental analysis (NCA) with linear trapezoidal rule was used to calculate pharmacokinetics parameters using R statistical software version 4.0.2^35^. The primary and secondary pharmacokinetic parameters for *R-*praziquantel, *S-*praziquantel and total praziquantel were calculated including maximum plasma concentration (C_max_) in ng/mL, time needed to reach C_max_ (T_max_) in hours, area under the concentration–time curve from 0 h to infinity (AUC_∞,_ ng × h/mL), area under the concentration–time curve from 0  to 8 h post-dose (AUC_8,_ ng × h/mL) and terminal half-life (t_1/2_) in hours. C_max,_ T_max_, t_1/2,_ AUC_8,_ and AUC_∞_ were directly calculated from the pharmacokinetics raw data using the PKNCA package version 0.9.4 implemented in R^36^.

### Statistical data analysis

Statistical data analysis was performed by R software. Results were summarized as mean ± standard deviations (SD) or median (interquartile range—IQR) for continuous variables and proportions for categorical variables. The log-transformed plasma concentrations and calculated AUC_8_ were dose-normalized by dividing the values with dose received by each participant. Pearson’s correlation coefficient (r^2^) was used to determine the correlation between praziquantel and its enantiomers plasma concentrations at each specific sampling time-point and AUC_8_. Sampling time-point with significantly highest r^2^ value than any other time-point was considered as an optimal single sampling time-point for monitoring of praziquantel bioavailability in children.

### Ethical approval

The study was reviewed and approved by the National Institute for Medical Research, Dar es Salaam, Tanzania (Ref. No. NIMR/HQ/R.8a/Vol.IX/2343), Muhimbili University of Health and Allied Sciences Institutional Review Board (Ref. No. 2016-5-25/AEC/Vol.X/03) and Stockholm Ethics Committee (Ref. No. 2020-00845). All participants received comprehensive information about the study and provided written informed consent (parents/guardians) and assent (eligible children) for participating in the study.

## Data Availability

All relevant data presented in this work are contained within the manuscript.
